# Prioritization in visual working memory enhances memory retention and speeds up processing in a comparison task

**DOI:** 10.1007/s10339-020-00967-7

**Published:** 2020-03-23

**Authors:** Christian H. Poth

**Affiliations:** Neuro-cognitive Psychology, Department of Psychology, and Cluster of Excellence Cognitive Interactions Technology, Bielefeld University, P.O. Box 100131, 33501 Bielefeld, Germany

**Keywords:** Visual cognition, Perception, Visual working memory, Retro-cue effect

## Abstract

Visual working memory retains visual information for controlling behavior. We studied how information in visual working memory is prioritized for being used. In two experiments, participants memorized the stimuli of a memory display for a brief interval, followed by a retro-cue. The retro-cue was either valid, indicating which stimulus from the memory display was relevant (i.e., had priority) in the upcoming comparison with a probe, or was neutral (uninformative). Next, the probe was presented, terminated by a mask, and participants reported whether it matched a stimulus from the memory display. The presentation duration of the probe was varied. Assessing performance as a function of presentation duration allowed to disentangle two components of working memory: memory retention and the speed of processing the probe for the memory-based comparison. Compared with neutral retro-cues, valid retro-cues improved retention and at the same time accelerated processing of the probe. These findings show for the first time that prioritization in working memory impacts on distinct mechanisms: retrospectively, it supports memory retention, and prospectively, it enhances perceptual processing in upcoming comparison tasks.

Visual working memory (VWM) is a cornerstone of human visual cognition. It temporarily retains visual information and makes it accessible for cognitive operation, report, and action control (Eriksson et al. 2015; Oberauer 2009; Poth and Schneider 2016a; Schneider 2013). VWM has only limited capacity (Luck and Vogel 1997; Shibuya and Bundesen 1988; Sperling 1960). Efficient use of this capacity dictates selectivity: currently relevant information must enter VWM with priority over less relevant information. This prioritization is performed by mechanisms of visual attention (Bundesen 1990; Bundesen et al. 2005; Duncan and Humphreys 1989; Schneider 1995). The bulk of attention research focused on prioritization up to the time of encoding into VWM (Bundesen et al. 2015; Duncan 2006; Poth and Schneider 2013). However, flexible visual cognition requires that changes of priority can be accommodated also when they happen after information has entered VWM.

Indeed, more recent research demonstrated that prioritization continues after encoding into VWM. This research made use of the retro-cuing paradigm (Griffin and Nobre 2003; Landman et al. 2003). Participants memorized a set of visual stimuli, the *memory display*, over a retention interval which was followed by a probe stimulus. The task was to report whether the probe matched an item from the memory display. A so-called *retro*-*cue* (i.e., a “retrodictive” cue) was shown after the memory display but before the probe appeared. In the experiments of current interest, retro-cues could be valid or neutral (Astle et al. 2012; Kuo et al. 2012). A valid retro-cue predicted which of the items from the memory display was going to be relevant for the upcoming comparison with the probe. A neutral retro-cue did not contain any predictive information regarding this comparison. The central result is that valid retro-cues improved comparison performance relative to neutral retro-cues. Over a decade of research accumulated evidence for beneficial effects of valid retro-cues in different versions of the basic paradigm (Astle et al. 2012; Griffin and Nobre 2003; Landman et al. 2003; Makovski and Jiang 2007; Makovski et al. 2008; Souza et al. 2014). Thus, it seems safe to conclude that valid retro-cues prioritize an item from a preceding memory display, while the memory display is retained in VWM.

Still controversial, however, is the question which mechanisms underlie the prioritization within VWM (Souza and Oberauer 2016). Current accounts assume that valid retro-cues improve comparison performance by manipulating the representations of the memory display in VWM. Specifically, some authors propose that they strengthen the VWM representation of the cued item, increasing the utility of this item for the comparison (Kuo et al. 2011; Lepsien et al. 2011; Nobre et al. 2008). Others propose that they free VWM capacity and reduce interference within VWM by having uncued items removed from VWM (Souza et al. 2014; Williams et al. 2013). Again others suggest that valid retro-cues protect the cued item against decay (Matsukura et al. 2007; Murray et al. 2013; Pertzov et al. 2013) or new interfering information (such as from the probe; Makovski et al. 2008; Makovski and Jiang 2007). Finally, some suggest that valid retro-cues grant the cued items priority in the process of being compared to the probe (Astle et al. 2012; Makovski et al. 2008; Nobre et al. 2008). Fundamental to all these accounts is that retro-cues are, as the term implies, retroactive. That is, all accounts assume that valid retro-cues engage mechanisms that, in one way or the other, prioritize information from the past which is now retained in VWM.

Here, we ask whether retro-cues facilitate memory retention in VWM, or whether they enhance the future perceptual processing in service of the comparison task, or both. To this end, we introduce a novel paradigm which allows to disentangle such retrospective and prospective effects of retro-cues (Fig. 1). Participants briefly viewed a memory display of two colored squares and memorized them over a retention interval. This interval outlasted iconic memory traces and thus called for retention in VWM (for a review, see Irwin and Thomas 2008). Afterward, a probe stimulus was presented that either matched or did not match an item from the memory display, with each alternative occurring in half the trials. The probe was presented at a location different from the stimuli of the memory display. This ensured that comparisons of probe and memorized stimuli needed to rely on VWM, as opposed to more fragile location-specific forms of short-term memory (see Pinto et al. 2013). Participants’ task was to indicate whether or not the probe matched an item from the memory display. A retro-cue appeared after the retention interval but before the probe. The retro-cue was either valid or neutral. A valid retro-cue pointed at the location of one of the items from the preceding memory display, the one that was going to be relevant for the upcoming comparison with the probe. Specifically, if the probe matched an item from the memory display, it was always the one indicated by the retro-cue. A neutral retro-cue did not contain any specific location information. We varied the presentation duration of the probe and terminated it with a pattern mask. This enabled us to assess performance as a function of the presentation duration. To disentangle the retrospective and the prospective effects of retro-cues, we fit these data with an exponential model (cf. Bundesen 1990; Wickelgren 1977) and compared the estimated parameters between valid and neutral retro-cues. The exponential model comprised three parameters. First, the level of asymptotic performance which is reached when the probe is shown long enough (see the upper asymptotes of the smooth curves in Fig. 2). The perceptual processing of the probe should improve with increasing presentation duration (e.g., Petersen and Andersen 2012; Shibuya and Bundesen 1988). At the asymptote, however, performance stops to increase with increasing presentation duration of the probe. Therefore, when the asymptote was reached, perceptual processing (encoding) of the probe should be over and variations of the asymptote should be due to post-perceptual factors. In the present case, variations in the asymptote should reflect the performance level for retaining the items of the memory display in VWM. Second, the rate at which performance increases with increasing presentation duration of the probe toward asymptotic performance (see how steeply the smooth curves increase with increasing presentation duration in Fig. 2). This is a well-established measure of processing speed (Bundesen 1990; Bundesen et al. 2015; Shibuya and Bundesen 1988; Wickelgren 1977). In contrast to reaction times, this measure has the important advantage that it reflects the specific perceptual (or cognitive) process without contributions of processes such as response selection or motor execution (that are always included in reaction times, see Finke et al. 2005, for a related discussion). Applied to the present case, the measure of processing speed should refer to the speed with which the probe is perceptually processed in order to accomplish the comparison with the items of the memory display. Third, a temporal threshold, reflecting the presentation duration of the probe that must be exceeded to increase performance above chance level (cf. Bundesen 1990; Wickelgren 1977).

**Fig. 1:**
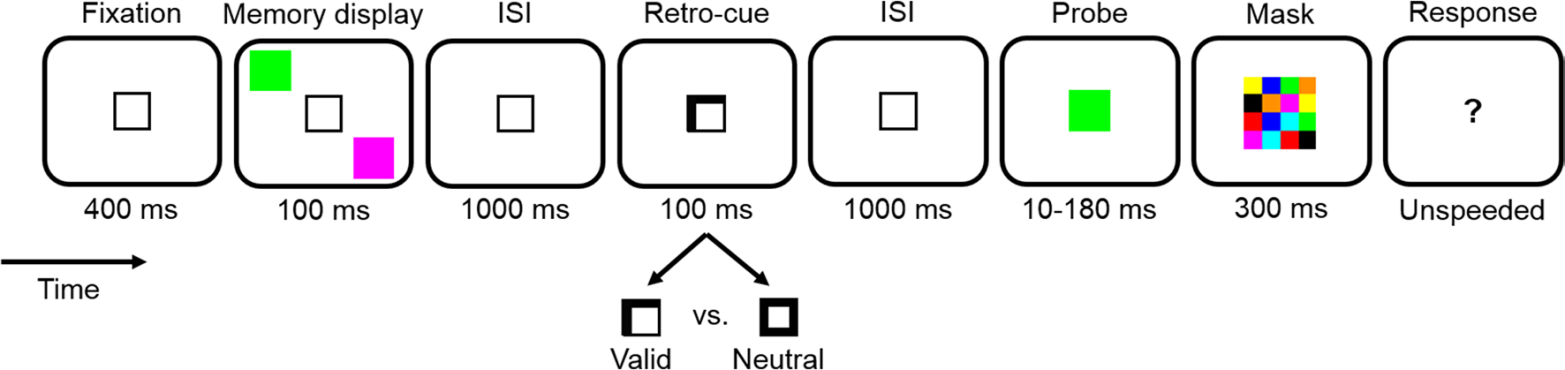
Paradigm of Experiment A. The paradigm of Experiment B was similar but differed in the display durations (and other aspects, see the Methods). Participants fixated a fixation square, after which a memory display with two colored squares appeared (the squares appeared at two out of four possible positions, whereby each pairing occurred equally often). After an interstimulus interval (ISI), a retro-cue was shown. If the retro-cue was valid, it indicated the location of the previous item from the memory display that was going to be relevant for the current trial. If it was neutral, it did not contain any predictive information. After another ISI, a probe was presented whose presentation duration was parametrically varied across trials. The probe was terminated by a pattern mask. In the end of a trial, a question mark prompted participants to indicate whether or not the probe matched an item from the memory display

**Fig. 2:**
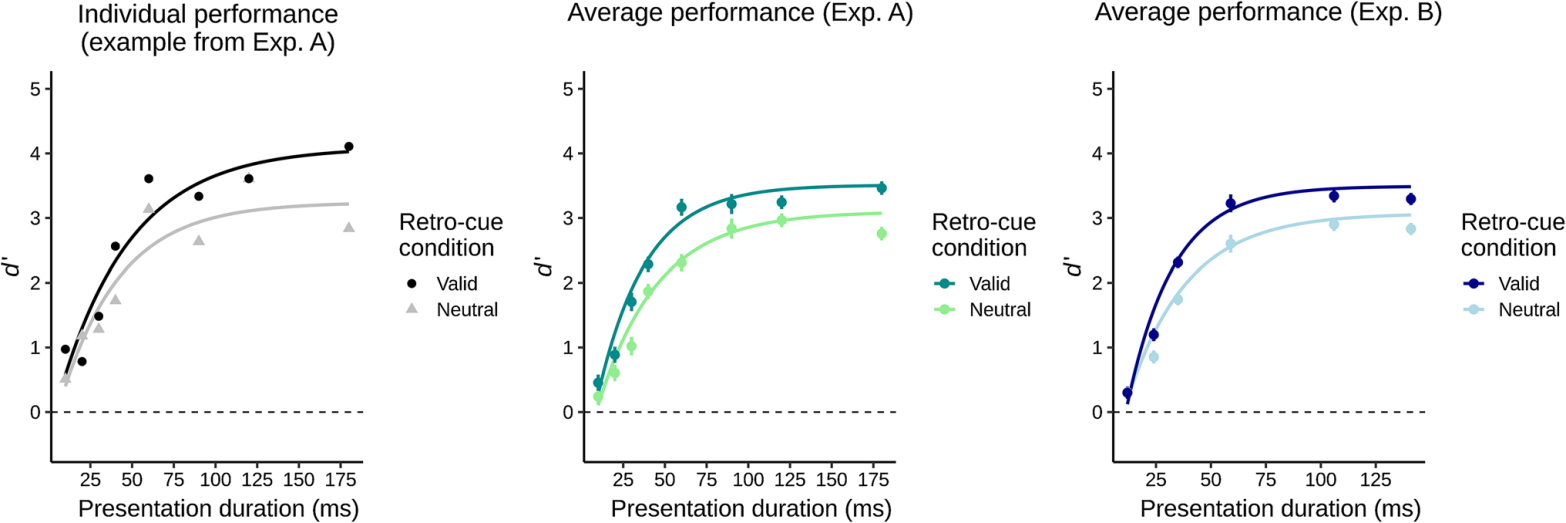
Performance of an individual participant of Experiment A (left) and average performance for the participants of Experiment A (middle) and B (right). Data points represent (average) performance (*d*′) in indicating whether or not probes matched an item from the memory display at each of the presentation durations of the probes. Error bars indicate ± one standard error for within-subjects designs (Loftus and Masson 1994). The two retro-cue conditions are depicted separately. Smooth curves represent least-squares fits of the exponential model to the data of the two retro-cue conditions. As model fitting was performed for each individual in each condition, the curves for the average performance result from averaging the parameters fitted to the data of individual participants

If retro-cues facilitate memory retention in VWM, then valid retro-cues should result in a higher asymptotic performance than neutral ones. In contrast, if retro-cues enhance processing for the upcoming comparison task, then valid retro-cues should lead to a higher processing speed of the probe than neutral ones. We first tested these hypotheses in Experiment A. Next, we performed Experiment B as a replication experiment further substantiating the results of Experiment A.

## Methods

### Participants

Eleven participants were paid to perform Experiment A. One additional participant was excluded because of problems with the eye tracker calibration (see below) and corrupted eye-tracking data files. The participants were between 21 and 34 years old (*MD* = 24 years), eight were female, three were male, and all reported normal or corrected-to-normal (contact lenses) visual acuity and normal color vision. Thirteen participants received course credit for performing Experiment B. They were between 18 and 27 years old (*MD* = 20 years), eleven were female, two male, and all reported normal or corrected-to-normal visual acuity and normal color vision. All participants gave written informed consent before participation; the experiments were approved by Bielefeld University’s ethics committee and complied with the ethical guidelines of the German Psychological Association (DGPs).

### Apparatus and stimuli

Both experiments took place in a dimly lit room. A chin-and-forehead rest (Experiment A) and a chin rest (Experiment B) ensured that participants viewed the CRT monitors from a distance of 71 cm. In Experiment A, the CRT monitor (G90fB, ViewSonic, Brea, CA, USA) ran at a refresh rate of 100 Hz and a resolution of 1024 × 768 pixels (corresponding to physical dimensions of 36 × 27 cm). For control purposes, a video-based desktop-mounted eye tracker sampled participants’ right eyes at 1000 Hz (Eyelink 1000, SR Research, Mississauga, Ontario, Canada; 9-point grid calibration). Experiment B did not use eye tracking, because it was not available in the employed laboratory setup. In Experiment B, the CRT monitor (Trinitron MultiScan G420, Sony, Park Ridge, NJ, USA) ran at a refresh rate of 85 Hz and a resolution of 1024 × 768 pixels (corresponding to physical dimensions of 36 × 27 cm). Responses were collected using standard computer keyboards (QWERTZ layout). The experiments were controlled by the Psychophysics toolbox (3.0.12; Kleiner et al. 2007; Pelli 1997, and in Experiment A, the Eyelink toolbox, 3.0.12; Cornelissen et al. 2002) extensions for Matlab (R2014b in Experiment A, R2013b in Experiment B, The MathWorks, Natick, MA, USA).

Stimulus luminance was measured using an LS-110 luminance meter (MINOLTA, Osaka, Japan). Stimulus luminance is reported for Experiments A and B side by side (i.e., luminance A/luminance B). An empty light-gray square was used as fixation stimulus (0.67 × 0.67° of visual angle, with a linewidth of 4 pixels; 59/45 cd/m^2^), henceforth called fixation square. Valid retro-cues consisted in a brightening of two lines of this square, neutral ones in a brightening of the whole square (102/114 cd/m^2^). The stimuli of the memory display were squares (0.67 × 0.67°) of the following eight colors: red (RGB: 255, 0, 0; 34/23 cd/m^2^), magenta (RGB: 255, 0, 255; 43/34 cd/m^2^), yellow (RGB: 255, 255, 0; 112/101 cd/m^2^), orange (RGB: 255, 145, 0; 59/44 cd/m^2^), blue (RGB: 0, 0, 255; 17/12 cd/m^2^), cyan (RGB: 0, 255, 255; 103/92 cd/m^2^), green (RGB: 0, 255, 0; 100/80 cd/m^2^), and black (RGB: 0, 0, 0; 1/< 1 cd/m^2^). For each individual participant, 99 pattern masks were algorithmically created in the beginning of the experiment. Masks consisted of a square composed of a 4 × 4 matrix of smaller squares (0.30° × 0.30° each) whose colors were randomly chosen from the set of colors with the constraint that each color occurred twice in each mask (see Fig. 1 for an example mask). The gray background had a luminance of 34/22 cd/m^2^. The white question mark of the response screen was written in Arial (0.50° × 1.00°; 102/114 cd/m^2^).

### Procedure and design

Figure 1 illustrates the paradigm of Experiment A (the paradigm of Experiment B was similar in most respects, see below). Participants started each trial by pressing the space bar. In the beginning of a trial, the fixation square was shown at screen center for 400 ms. In Experiment A, the eye tracker monitored if eyes were open (i.e., pupils visible) until the end of this fixation period, and if they were not, the period was prolonged until the next screen refresh after the eyes were open again. In Experiment B, eye behavior was not recorded. The fixation square stayed on for the most of a trial. After the fixation period, the memory display containing two differently colored squares was shown for 100 ms in Experiment A and for 94 ms in Experiment B. The colors of the squares were randomly chosen from the set of used colors. The squares appeared at two out of four possible positions (2° from screen center horizontally to the left or right × vertically to the left or right, see Fig. 1), and this was randomized across trials with each pairing of positions occurring equally often. The memory display was followed by an interstimulus interval (ISI) of 1000 ms, after which the retro-cue was presented for 100 ms in Experiment A and 94 ms in Experiment B. Valid retro-cues consisted in a selective brightening of two sides of the fixation square, forming an arrow pointing at one of the two locations of the squares of the previous memory display. Across trials, each location was cued equally often. Neutral retro-cues consisted in a brightening of the whole fixation square. The retro-cue was followed by another ISI of 1000 ms. The probe then replaced the fixation square at screen center. In Experiment A, the probe was shown for eight different durations (10, 20, 30, 40, 60, 90, 120, and 180 ms). In Experiment B, it was shown for six different durations (12, 24, 35, 59, 106, and 141 ms). The presentation of the probe was terminated by a central pattern mask lasting for 300 ms in Experiment A and 306 ms in Experiment B. Afterward, a central question mark was presented until participants responded. Participants were instructed to respond with the F9-key if the probe matched an item from the memory display and the F1-key if it did not match any of them. There was no time limit for the response. On half of the trials, the color of the probe matched the color of one of the items from the memory display. On the other half, it had one of the colors that did not appear on this trial. Participants were informed that if a valid retro-cue was shown and the probe matched the color of an item from the memory display, then this would be the color of the item indicated by the retro-cue.

Participants performed Experiment A in a single session of 768 trials, 48 trials per retro-cue condition (valid vs. neutral) and per presentation duration of the probe. Participants performed Experiment B in two sessions (on separate days) of 576 trials each, yielding 1152 trials in total, 96 trials per retro-cue condition and per presentation duration of the probe. Trials were administered in randomized order in Experiment A and in both sessions of Experiment B. Participants performed 30 training trials (randomly chosen with replacement from all trial types) before each participation.

## Results

To control for response biases, performance in indicating whether the probe matched an item from the memory display was assessed as *d*′ (the *z*-transformed rate of “yes”-responses to probes matching an item from the memory display minus the *z*-transformed rate of “yes”-responses to probes not matching an item from the memory display; 0.5 was added to the data cells on which rates were computed to prevent infinite values of *d*′, see Macmillan and Creelman 2005). Performance was assessed as a function of presentation duration of the probe. For each participant and each retro-cue condition, these data were fit with an exponential model of the type

$$d^{\prime } = \omega \left( {1 - \exp \left( { - v*\left( {t - t_{0} } \right)} \right)} \right)$$

where *ω* is the upper asymptote of performance (in $d^{\prime }$), *v* is the rate parameter of the exponential distribution which measures processing speed of the probe within the comparison with the items of the memory display (in items/s; cf. Bundesen 1990; Bundesen et al. 2015; Wickelgren 1977). The third parameter, *t*_0_, is a temporal threshold consisting in the presentation duration of the probe that has to be exceeded to increase performance above chance level (in ms; cf. Bundesen 1990). Fitting was accomplished using the nonlinear least-squares (nls) method implemented in R (3.6.3; R Core Team 2020). Figure 2 depicts the performance in each retro-cue condition and corresponding model fits, for one participant in Experiment A and for the average data of both experiments. Table 1 provides descriptive statistics of estimated parameters for the two retro-cue conditions of both experiments. Goodness-of-fit was quantified as Pearson’s correlation *r* between the predicted values based on the fitted model and participants’ observed values (see Table 1).

**Table 1:** Descriptive statistics of Experiments A and B

	Experiment A		Experiment B	
	Valid retro-cue	Neutral retro-cue	Valid retro-cue	Neutral retro-cue
ω	3.51 (0.50)	3.10 (0.64)	3.49 (0.81)	3.07 (0.89)
*v*	34.70 (11.08)	27.80 (10.53)	47.42 (20.22)	36.72 (14.72)
*t*_0_	8 (3)	9 (5)	11 (3)	10 (3)
*r*	.945 (.030)	.917 (.036)	.962 (.027)	.961 (.029)

Descriptive statistics of estimated parameters for the two retro-cue conditions of Experiments A and B. Provided are means and standard deviations (in parentheses) across participants for the asymptotic performance level *ω* (in *d*′), the processing speed *v* (in items/s), the temporal threshold *t*_0_ (in ms), and for Pearson’s correlation between the values predicted by the fitted model and the observed values

**Fig. 3:**
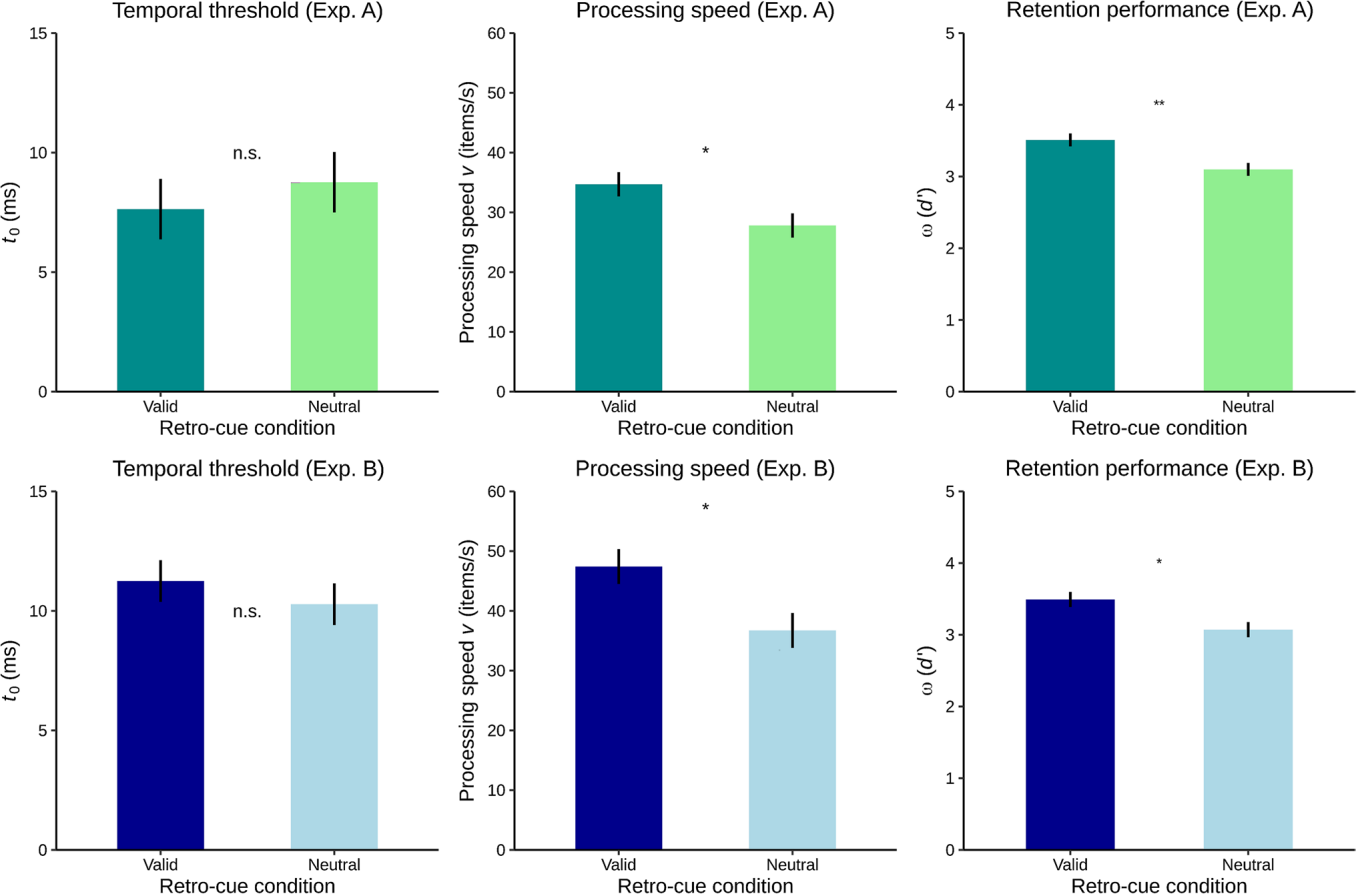
Estimated model parameters in the two retro-cue conditions of Experiment A (upper panel) and Experiment B (lower panel). Depicted are the means of the retention performance ω (in *d*′), of the processing speed *v* (in items/s), and of the temporal threshold *t*_0_ (in ms), across participants. Error bars indicate ± one standard error for within-subjects designs (Loftus and Masson 1994). n.s. nonsignificant. *p <.05. **p < .01

The parameter estimates were compared between the retro-cue conditions using paired-samples *t* tests (two sided, with a significance criterion of *p* < .05, and *d*_*z*_ as effect size Cohen 1988, and for which the assumption of normally distributed differences was assessed beforehand using Shapiro–Wilk tests). *t* tests were supplemented with corresponding Bayes Factors (BF_10_, Rouder et al. 2009, whereby values greater than one favor the alternative and values smaller than one favor the null hypothesis). Figure 3 illustrates the parameter comparisons between the retro-cue conditions of the two experiments.

In Experiment A, the retention performance ω was significantly higher when retro-cues were valid than when they were neutral, *t*(10) = 3.212, *p* = .009, *d*_z_ = 0.97, BF_10_ = 6.42. Experiment B replicated this result, *t*(12) = 2.808, *p* = .016, *d*_*z*_ = 0.78, BF_10_ = 3.99. These findings demonstrate a retrospective effect of retro-cues. They indicate that valid retro-cues facilitated the retention of the relevant item from the memory display as compared with neutral retro-cues.

The processing speed *v* was significantly higher when retro-cues were valid compared with neutral, and this was likewise the case in Experiment A, *t*(10) = 2.400, *p* = .037, *d*_z_ = 0.72, BF_10_ = 2.14, and Experiment B, *t*(12) = 2.589, *p* = .024, *d*_*z*_ = 0.72, BF_10_ = 2.89. These findings reveal a prospective effect of retro-cues on the future visual processing. Specifically, compared with neutral retro-cues, valid retro-cues increased the speed with which an upcoming probe was processed, in order to assess whether it had been contained in the preceding memory display.

There were no differences between the valid and the neutral retro-cue condition regarding the temporal threshold, *t*_0_, neither in Experiment A, *t*(10) = − 0.629, *p* = .543, *d*_*z*_ = − 0.19, BF_10_ = 0.35, nor in Experiment B, *t*(12) = 0.787, *p* = .447, *d*_*z*_ = 0.22, BF_10_ = 0.36.

Taken together, the findings of Experiment A and the replication Experiment B converged closely. This was further substantiated by mixed (within between) analyses of variances that included the experiment as a between-factor. For none of the three parameters, the effect of retro-cue condition (valid vs. neutral) and the effect of experiment (A vs. B) interacted, all *Fs*(1, 22) < 1, all *p*s > .33. For the retention performance, the absolute values did not differ between the two experiments, *F*(1, 22) = .007, *p* = .936. For the temporal threshold, the absolute values differed between the experiments, *F*(1, 22) = 5.533, *p *= .028 (see Fig. 3). For the processing speed, the difference between the absolute values in the experiments approached significance, *F*(1, 22) = 3.807, *p* = .064. It is important to note, however, that these between-subjects differences do not conflict with the findings of interest, namely the within-subjects effects of valid versus neutral retro-cues. Indeed, the between-subjects differences in temporal threshold and processing speed are not surprising, because the two parameters have previously been shown to be subject of considerable interindividual variation (see, for example, the individual model curves in Poth and Schneider 2018 and Poth et al. 2018).

## Discussion

The present experiments reveal that retro-cues impact on two distinct components of VWM performance. First, valid retro-cues improve memory retention in VWM. This was evident from the higher retention performance when retro-cues were valid compared with neutral. We assessed retention performance as the asymptote of performance as a function of the presentation duration of the probe. Asymptotic performance reflects a component of VWM performance that is independent from perceptual processing of the probe, because performance does not improve further when the probe is presented longer. Second, valid retro-cues enhanced the speed with which probe stimuli were perceptually processed in order to be compared to the items retained in VWM. We assessed the speed of processing the probe for this memory-based comparison as the rate of performance increase with increasing presentation duration of the probe.

Valid retro-cues improved memory retention. This indicates that prioritization modulates representations in VWM independently of the time available to process the probe for the upcoming comparison task. This is in line with several not mutually exclusive accounts assuming that retro-cues impact on VWM-based performance by directly modulating VWM representations. Valid retro-cues may strengthen the representations of cued items in VWM (Kuo et al. 2011; Lepsien et al. 2011; Nobre et al. 2008). They may remove uncued items from VWM, thereby freeing capacity and reducing interference within VWM (Souza et al. 2014; Williams et al. 2013). They may protect the representations of cued items in VWM against decay (Matsukura et al. 2007; Pertzov et al. 2013) or interfering new information (Makovski et al. 2008; Makovski and Jiang 2007).

Crucially, valid retro-cues also accelerated the perceptual processing of probe stimuli for performing the upcoming comparison task. This indicates that prioritization in VWM has effects beyond pure memory retention. In this way, this present finding calls for an extension of current accounts of the beneficial effects of valid retro-cues on VWM-based performance. The finding can be interpreted in two ways.

The first explanation is that the presentation duration of the probe stimulus determined the quality of its representation in VWM. The rate of performance increase with increasing probe presentation duration was higher when retro-cues were valid than neutral. Thus, valid retro-cues may have compensated for the low representational quality of the probe at short presentation durations, for example, by enhancing the VWM representations of the cued items, which would be in line with previous accounts (e.g., Kuo et al. 2011; Lepsien et al. 2011; Nobre et al. 2008). The present findings would then show that valid retro-cues improve a component of VWM that can be traded-off for the representational quality of the probe stimulus. However, with the effects on memory retention, the findings would also indicate that valid retro-cues improve a VWM component beyond this, one that does not interact with the representational quality of the probe.

The second explanation assumes that the acceleration of the processing of the probe resulted from a prospective monitoring process. A valid retro-cue indicates which of the items in VWM will be relevant for the comparison to an upcoming probe. Consequently, the environment may be monitored for the features of the cued item already before the probe appears. It has previously been shown that monitoring processes rely on visual attention (Poth et al. 2014). In the present case, the features of the cued item may be monitored for by engaging the pigeonholing (“attention to features”) mechanism from Bundesen’s theory of visual attention (TVA; Bundesen 1990; Bundesen et al. 2015). Pigeonholing influences the speed with which visual features of objects are processed for being encoded into VWM. This should happen by up- or down-regulating a perceptual bias for categorizing any given object as having a certain feature. The perceptual bias is internal, meaning that it is independent of what objects are actually viewed. Specifically, the perceptual bias for a certain feature may be implemented by increasing or decreasing the firing rates of visual neurons preferentially coding for this feature (Bundesen et al. 2005). Increasing the perceptual bias for the features of the cued item would increase the speed of encoding the probe into VWM, if the probe has these features. This would imply that the present processing acceleration stemmed from the trials on which the probe matched the cued item. Indeed, effects of retro-cues have been found more pronounced for such matches (Lepsien et al. 2005; Nobre et al. 2008). However, a processing acceleration on trials on which the probe did not match an item from the memory display could still be explained in terms of pigeonholing by assuming an additional decision deadline (cf. Bundesen’s 1990 explanation of target-absent response times in visual search). In this scenario, valid retro-cues would lower the deadline for processing the probe in order for deciding that it did not match an item from the memory display.

In sum, valid retro-cues offered benefits for VWM performance both retrospectively (for retention) and also prospectively (for perceptual processing). To further investigate the mechanisms by which retro-cues achieve this, future studies could include invalid retro-cues that indicate an item that would not be probed afterward. In this way, the studies could contrast beneficial effects of valid (vs. neutral) retro-cues with detrimental effects of invalid (vs. neutral) retro-cues.

Based on recent research, one might speculate whether active or passive working memory processes underlay the effects of valid retro-cues. Both types of processes rely on the sensory recruitment hypothesis, stating that the retention of information in VWM is enacted by the same visual brain areas that encode this information at first (Ester et al. 2013; Miller et al. 1996; Serences et al. 2009; Supèr et al. 2001). The hypothesis of active working memory processes relies on the assumption that retention in VWM is performed by maintaining the spiking activity of the neurons coding for the retained items and their features (Chelazzi et al. 1993). The retrospective effects of valid retro-cues on memory retention in VWM could consist in an increase in activity in neurons coding for the features of the cued item, or a decrease in those coding for features of other items, or both (Lepsien et al. 2011; Trapp and Lepsien 2012). An increase in spiking activity of these neurons could at the same time provoke the prospective effects of valid retro-cues. The increased firing would support the future encoding of stimuli by these neurons, explaining why processing of the probe was accelerated in the present experiments.

The hypothesis of passive working memory processes is grounded on recent evidence questioning maintained spiking activity as the sole neuronal implementation of retention in VWM (for an overview, see Stokes 2015). Findings from single-cell neurophysiology (Stokes et al. 2013) and brain imaging and stimulation (e.g., Rose et al. 2016; for a review, see Larocque et al. 2014) suggest a passive (V)WM (Schneider 2013), which presumably relies on increased synaptic connectivity rather than maintained spiking activity (Mongillo et al. 2008; though synaptic connectivity may stem from an initial increase in neuronal firing, e.g., Stokes 2015). Valid retro-cues could also exert their effects here, by modulating the synaptic connectivity in visual brain areas at short time scales. Valid retro-cues could increase the synaptic connectivity of neurons coding for the features of the cued item. Enhanced synaptic connectivity could make VWM representations more robust, offering another explanation why valid retro-cues improved retention performance. Interestingly, if valid retro-cues increased the short-term synaptic connectivity of these neurons, this might also increase their efficiency of encoding new stimuli (Stokes et al. 2013; Sugase-Miyamoto et al. 2008). Hence, this provides another explanation of why valid retro-cues accelerated processing of probes. It is important to note, however, that both active and passive working memory processes are feature based. Therefore, both processes would only be able to operate if the features of probes matched the features of the cued items in VWM. This would call for an additional process implementing a decision deadline, in the same way as the prospective monitoring process that relied on Bundesen’s (1990) pigeonholing mechanism.

To conclude, the present study shows that priority within VWM not only affects the retention of past information but also future processing in a comparison task. Visual attention seems not only to set processing priorities for encoding into VWM (Bundesen 1990) and for selection within VWM (Griffin and Nobre 2003; Landman et al. 2003), but at the same time also for processing upcoming information. In this vein, visual attention may provide a bridge between episodes of visual processing that are composed of encoding into and retention in VWM (as proposed by Schneider 2013; see also Poth et al. 2015; Poth and Schneider 2016b).
